# Post-activation Potentiation Versus Post-activation Performance Enhancement in Humans: Historical Perspective, Underlying Mechanisms, and Current Issues

**DOI:** 10.3389/fphys.2019.01359

**Published:** 2019-11-01

**Authors:** Anthony J. Blazevich, Nicolas Babault

**Affiliations:** ^1^School of Medical and Health Science, Centre for Exercise and Sports Science Research (CESSR), Edith Cowan University, Joondalup, WA, Australia; ^2^Faculty of Sport Sciences, French National Institute of Health and Medical Research (INSERM), Unit 1093 Cognition, Action and Sensorimotor Plasticity, Centre for Performance Expertise, University of Burgundy and Franche-Comté, Dijon, France

**Keywords:** myosin phosphorylation, neuromuscular, warm-up, muscle, temperature, methodology, performance

## Abstract

Post-activation potentiation (PAP) is a well-described phenomenon with a short half-life (~28 s) that enhances muscle force production at submaximal levels of calcium saturation (i.e., submaximal levels of muscle activation). It has been largely explained by an increased myosin light chain phosphorylation occurring in type II muscle fibers, and its effects have been quantified in humans by measuring muscle twitch force responses to a bout of muscular activity. However, enhancements in (sometimes maximal) voluntary force production detected several minutes after high-intensity muscle contractions are also observed, which are also most prominent in muscles with a high proportion of type II fibers. This effect has been considered to reflect PAP. Nonetheless, the time course of myosin light chain phosphorylation (underpinning “classic” PAP) rarely matches that of voluntary force enhancement and, unlike PAP, changes in muscle temperature, muscle/cellular water content, and muscle activation may at least partly underpin voluntary force enhancement; this enhancement has thus recently been called post-activation performance enhancement (PAPE) to distinguish it from “classical” PAP. In fact, since PAPE is often undetectable at time points where PAP is maximal (or substantial), some researchers have questioned whether PAP contributes to PAPE under most conditions *in vivo* in humans. Equally, minimal evidence has been presented that PAP is of significant practical importance in cases where multiple physiological processes have already been upregulated by a preceding, comprehensive, active muscle warm-up. Given that confusion exists with respect to the mechanisms leading to acute enhancement of both electrically evoked (twitch force; PAP) and voluntary (PAPE) muscle function in humans after acute muscle activity, the first purpose of the present narrative review is to recount the history of PAP/PAPE research to locate definitions and determine whether they are the same phenomena. To further investigate the possibility of these phenomena being distinct as well as to better understand their potential functional benefits, possible mechanisms underpinning their effects will be examined in detail. Finally, research design issues will be addressed which might contribute to confusion relating to PAP/PAPE effects, before the contexts in which these phenomena may (or may not) benefit voluntary muscle function are considered.

## Introduction

Both acute and chronic increases in physical function are goals of practitioners in both athletic and clinical arenas. Chronic improvements result from the longer term implementation of training and treatment methods, usually using a strategy of exercise periodization (Bompa and Haff, 1994). Acute improvements, however, may be evoked by the use of a wide variety of physical or psychological strategies during (or immediately before) training sessions or athletic (sporting) competitions.

With respect to acute performance enhancements, much attention has been given to the possibility that performance improvement can be achieved through strategies that induce a post-activation potentiation (PAP) in the working muscles. PAP has been defined as an enhanced muscle contractile response for a given level of stimulation following an intense voluntary contraction, which is measured as the maximum twitch force evoked by supramaximal electrical stimulation (Ramsey and Street, 1941; MacIntosh et al., 2012). However, the term “PAP” has more recently been used to describe a voluntary force or power enhancement after a high-intensity exercise-based warm-up, without confirmation by twitch stimulations that PAP was evoked and therefore that other factors that impact muscle function (e.g., muscle temperature, activation level/learning) do not underpin the enhancement. That is, it is rarely established that the muscle performance enhancement results from mechanisms that underpin “classical” PAP (e.g., phosphorylation of the myosin light chain; described in detail below). Thus, the question remains as to whether the voluntary performance enhancements seen after brief, high-intensity bouts of physical activity result from the same mechanisms that underpin PAP, or whether other changes that are commonly observed during warm-up activities better explain the enhancement.

Of additional importance is that studies examining acute performance enhancements commonly use a similar experimental design:

Performance Test 1 → Conditioning contraction(s) → Rest period → Performance Test 2 (and often Tests 3, 4, 5…).

Considering that the persistence of PAP (as classically defined) is significant for only a few minutes [usually <3 min; half-life ~28 s according to Vandervoort et al. (1983)] and yet the peak voluntary performance enhancement often occurs 6–10 min after the conditioning activity in most studies (Wilson et al., 2013), questions arise as to whether PAP coincides temporally with the acute voluntary performance enhancement seen after intense contractions in humans. Yet many studies do observe an enhancement of voluntary performance at Test 2 relative to Test 1, so the conditioning activity clearly has a practically meaningful effect. For these reasons, the term post-activation performance enhancement (PAPE) has recently been proposed for use (Cuenca-Fernandez et al., 2017) when a high-intensity voluntary conditioning contraction(s) leads to enhancement in voluntary muscular performance in a subsequent test without confirmatory evidence of classical PAP (i.e., twitch force assessment).

In order to explore the effects of acute bouts of physical activity on muscle function during both electrically evoked (used to test “classical” PAP effects) and voluntary tests, the aims of the current review were to: (1) provide an historical perspective of the evolution of PAP research, (2) critically evaluate the existing literature with respect to the mechanisms that might enhance functional performance or the muscle’s contractile response following a conditioning contraction (i.e., PAP/PAPE), (3) consider the potential functional implications of PAP/PAPE in both athletic and clinical populations, and (4) briefly discuss research study design limitations that need to be considered in future studies.

## A Brief History of Post-Activation Potentiation

**Figure 1:**
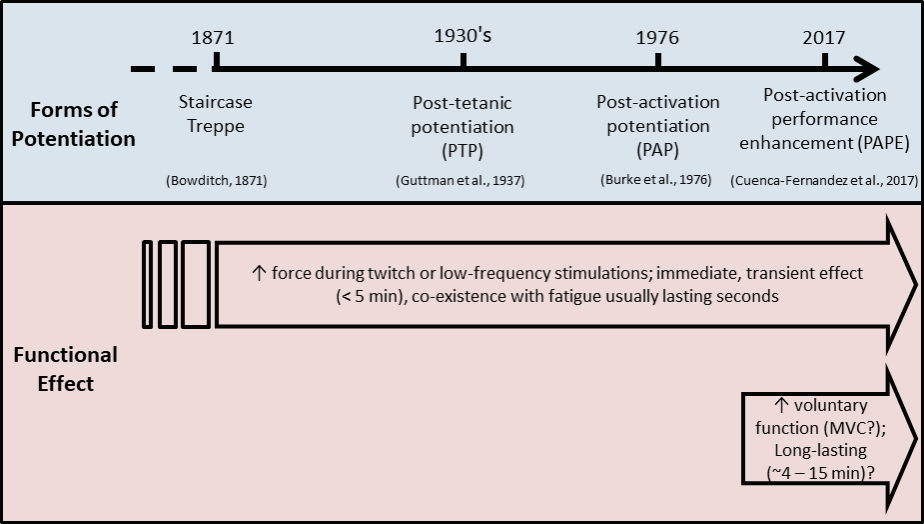
Many forms of potentiation have been described in human (and other) muscles (top bar). Staircase and treppe, post-tetanic potentiation, and post-activation potentiation have been well described and are observed as an increase in forces produced during muscle twitch (or low-frequency tetanic) stimulations. Post-activation performance enhancement has been studied more recently (our definition was first used in Cuenca-Fernandez et al., 2017), and is observed as an increase in voluntary [sometimes maximal (MVC)] muscle force, and has a different time course of action compared to other forms of potentiation (bottom bar).

Reports of the potentiation of a muscle’s contractile response following a conditioning contraction date back to the 19th century (Figure 1). Potentiation has traditionally been measured as an increase in the force of an isometric (i.e., fixed end) muscle twitch contraction that is transiently increased after a conditioning contraction (Ramsey and Street, 1941). There are three forms of activity-dependent potentiation: staircase, post-tetanic potentiation, and PAP. In all cases, detection of an enhanced contractile response relies on the application of a known stimulus to the muscle or motor nerve before and after a conditioning contraction. Activity-dependent potentiation is generally defined as an increased contractile response for a given stimulation that is dependent on contraction history, i.e., a prior muscle activation (Gossen and Sale, 2000; MacIntosh and Rassier, 2002). An enhanced response is observed after the conditioning contraction, which reaches a peak within seconds and subsequently decreases in amplitude over the next several minutes. The primary difference between the three forms of potentiation relates to the conditioning contraction eliciting the enhanced response: in staircase, the conditioning contractions comprise repeated, low-frequency electrical stimulations where contractions sequentially increase in amplitude; in post-tetanic potentiation, a brief high-frequency train of electrical stimulation acts as the conditioning contraction; in PAP, the enhanced contractile response (muscle twitch) is evoked by voluntary muscle activation (although tetanic stimuli may be used in animal models examining mechanisms underpinning potentiation responses).

Although it is difficult to locate the scientific origins of these different phenomena, it appears that “staircase” or “treppe” is the oldest term used. In the paper of Lee (1907), these terms signified “the fact that repeated responses of a tissue to repeated and equal stimuli increase for a time in intensity.” This implies a progressive increase in twitch contractile response during repeated low-frequency stimulations (Colomo and Rocchi, 1965; Isaacson, 1969), although this phenomenon was known earlier and originated from older pioneering works. Indeed, the term “treppe” was first introduced by Bowditch (1871) while investigating cardiac frog muscle. But earlier, Ranke (1865) pointed out that “everyone knows that the first twitch of a muscle is not its greatest” but that its reasons were “wholly obscure.” Subsequently, Marey (1866) identified that the height of the muscle force-time curve during continued activity first increases and then decreases. Marey therefore linked potentiation and fatigue, which was subsequently well demonstrated by Brown and Tuttle and others (Brown and Tuttle, 1926; Guttman et al., 1937). The term “post-tetanic potentiation” appeared later, in the 1930’s (Guttman et al., 1937; Brown and Von Euler, 1938). It corresponded to an increase in amplitude of twitch tension after a sustained muscle tetanic stimulation, generally at a high stimulation frequency. This potentiating effect was found to be independent of the integrity of the neuromuscular transmitting apparatus (Brown and Von Euler, 1938). Thus, in these early works, the terms used for potentiating effects described phenomena residing exclusively in the muscle and could not be influenced by mechanisms of central drive.

The origin of the term “post-activation potentiation” (PAP) is more difficult to pinpoint. Burke et al. (1976), however, used this term in the 1970’s as a distinction from post-tetanic potentiation or staircase potentiation; they referred to repetitive activation at frequencies and with numbers of pulses compatible with natural activation. This was considered a more general way to characterize “activity-dependent potentiation” since it referred specifically to an augmentation of twitch tension that followed any type of repetitive activation. It is now commonly accepted that PAP is induced by voluntary activation of the muscle (at maximal or near-maximal intensities) whereas post-tetanic potentiation is induced by an involuntary (electrical) tetanic stimulation (Sale, 2002; MacIntosh et al., 2012); either way, PAP is verified by an increase in the peak twitch force of muscle.

**Figure 2:**
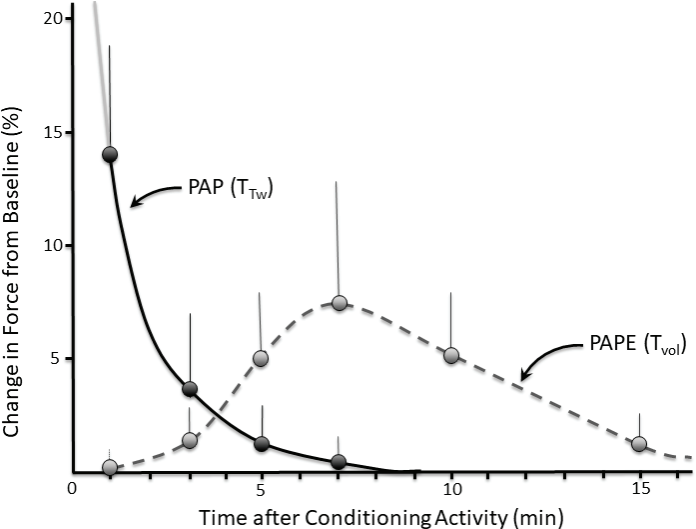
Time course (with example error bars) of post-activation potentiation (PAP) measured as the torque evoked during a twitch contraction (T_Tw_) versus post-activation performance enhancement (PAPE) measured as the torque produced during a voluntary contraction (T_Vol_). PAP and PAPE exhibit different temporal profiles, suggesting that they are at least partly different phenomena. Data for illustrative purposes only; experimental data can be seen in Seitz et al. (2015); gray solid line indicates that PAP would be higher at time points < 1 min (>80% according to Hamada et al., 2000). Note: functions are plotted from 1 min because the first experimental data were collected at this time point.

The exploration of increases in twitch torque in response to brief maximal voluntary contractions (MVC) was popularized by the publication of several important papers in the early 1980’s (Belanger and Quinlan, 1982; Belanger et al., 1983; Vandervoort et al., 1983). In these studies, the authors demonstrated that PAP was dependent upon the characteristics of the muscle under investigation, the duration and intensity of the conditioning contractions, and the muscle length. For instance, PAP appeared to be optimized after a 10-s MVC as opposed to shorter or longer activations, with longer contractions suppressing PAP through mechanisms causing “fatigue” (Vandervoort et al., 1983). PAP-induced peak twitch forces were observed to be more than twice as large as unpotentiated twitches, with the effect detected almost immediately after the conditioning contractions and decreasing dramatically to one-half after 28 s and then more slowly over the next 8–10 min (Vandervoort et al., 1983), as depicted in Figure 2. Thus, the effect was found to be short-lived and likely of potential practical benefit only in the first few minutes after being elicited.

**Figure 3:**
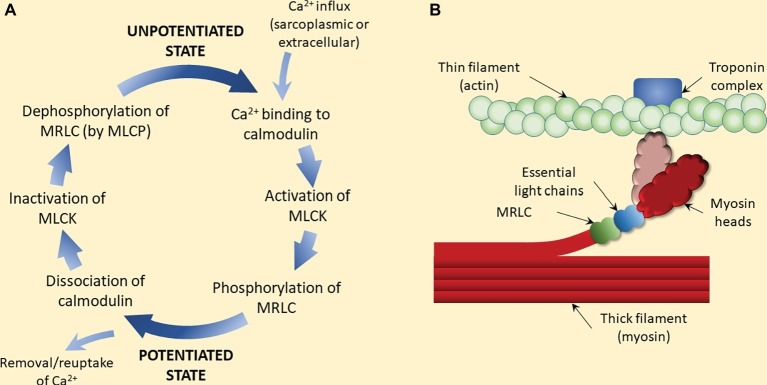
Proposed mechanism of (classic) post-activation potentiation (PAP). **(A)** Calcium ions activate myosin light chain kinase (MLCK) subsequent to calcium-calmodulin interaction. MLCK then phosphorylates the myosin regulatory light chain (MRLC), which is believed to trigger the rotation of the myosin head away from the thick filament (myosin backbone) toward the thin filament (light color myosin head in panel B). Removal or reuptake of calcium triggers the dissociation of calmodulin and inactivation of MLCK. Dephosphorylation of the MRLC by myosin light chain phosphatase (MLCP) completes the process of potentiation removal. **(B)** MRLC phosphorylation and subsequent rotation of the myosin head (light color) increases the probability of the head attaching to actin, and thus force production. At submaximal (i.e., below saturating) levels of calcium, this process increases force output at a given calcium concentration, i.e., calcium sensitivity. Since phosphorylation is rapid but dephosphorylation is relatively slow, a period of contractile activity (e.g., a warm-up period) is sufficient to augment contractile properties in subsequent contractions, even if a short period of rest is imposed (e.g., contractions are intermittent) (Manning and Stull, 1979).

The early classic studies located the origin of PAP within the muscles themselves, but the exact mechanism was elusive. It was eventually found that the (vertebrate) myosin II molecule of skeletal muscle contained a phosphorylatable light chain (Perrie et al., 1973), and it was subsequently hypothesized that this might provide a potential mechanism for PAP. Researchers demonstrated that post-tetanic potentiation persisted as long as the myosin light chain was phosphorylated (Manning and Stull, 1979). It was subsequently established that calcium-dependent myosin light chain phosphorylation rendered the actin-myosin complex more sensitive to calcium (Persechini et al., 1985), as illustrated in Figure 3. The consequence of myosin light chain phosphorylation was an increase in the rate of cross-bridge formation corresponding with a faster rate of force development (RFD) (Brown and Von Euler, 1938; Sweeney and Stull, 1990). It was also demonstrated that potentiation was most effective at low myoplasmic Ca^2+^ concentrations (Persechini et al., 1985) and would therefore more likely occur in twitch contractions or low-frequency tetanic contractions, but not contractions with a high (or maximal) stimulating frequency. The latter would produce saturating levels of Ca^2+^, thus preventing any impact of increased Ca^2+^ sensitivity. It was subsequently suggested (Sale, 2002) that brief maximal-effort voluntary contractions could also be potentiated because PAP is associated with an increase in peak RFD. In fact, an enhanced contractile response has been demonstrated for high-frequency (maximal) stimulations when contractions are very brief (MacIntosh et al., 2008).

More recently, the possibility that PAP might contribute to improved voluntary muscle force production in humans has been tested using movements that require maximal voluntary muscle activations, such as vertical jumping (Güllich and Schmidtbleicher, 1996; Gossen and Sale, 2000; French et al., 2003) and sprint running (McBride et al., 2005; Yetter and Moir, 2008; Winwood et al., 2016), cycling (Munro et al., 2017), and swimming (Hancock et al., 2015). According to the mechanisms of PAP presented above, PAP should enhance the RFD during MVC as well as submaximal force for a given level of neural activation, if they are brief (although see further discussion below), so performance enhancements should only result from increases of force rise at specific points in a jump, step, pedal, or stroke cycle when maximal (or near-maximal) forces are yet to be produced. Moreover, an increasing number of studies have evaluated muscular performance at a time when PAP should have dissipated, and most studies do not include an evaluation of the presence of PAP using electrically evoked twitches or low-frequency tetanic stimulations in order to determine its magnitude at the point of voluntary muscle testing. For this reason, it is not always clear whether “classic” PAP underpins the performance enhancement, and thus peripheral as well as central origins have been suggested for the increase in performance of maximal voluntary exercise performance *in vivo* (Güllich and Schmidtbleicher, 1996; Folland et al., 2008). Because the different time course of effect (e.g., Figure 2) as well as possible differences in the mechanisms underpinning force enhancement, Cuenca-Fernandez et al. (2017) recently proposed that the term “post-activation performance enhancement” (PAPE) should be used when high-intensity voluntary conditioning contraction(s) are performed with the intent of enhancing subsequent voluntary, rather than electrically evoked (twitch), force production. While this term has yet to be agreed for use in the scientific or clinical communities, a term such as this can be used to distinguish the changes in twitch force from voluntary force in these circumstances.

Consideration of this brief historical review leads to the conclusion that activity-dependent potentiation should be defined according to: (1) the conditioning contraction, i.e., whether it is electrically or voluntarily elicited (staircase vs. post-tetanic potentiation vs. PAP), and (2) the functional outcome, i.e., whether the enhancement of muscle function is measured using twitch contractions (PAP) or voluntary contractions (PAPE). However, at present these terms are rarely defined within the current literature.

For the remainder of the current paper, therefore, we will use the term “post-activation potentiation,” with the acronym PAP, to refer to increases in twitch forces evoked by prior muscle activity (but regardless of whether electrically stimulated or voluntary contractions are used to trigger the phenomenon), and we will use the term “post-activation performance enhancement,” with the acronym PAPE, to refer to increases in voluntary force production (or exercise performance) evoked by prior muscle activity. This distinction is important because, as will be discussed below, the mechanisms underpinning their effects, the time course of their effects, and the likely practical benefit derived from the phenomena may vary. Whatever the definition and underlying mechanisms, however, it is broadly agreed that intense muscle contractions may acutely improve force production in different populations and in different contraction situations, and thus understanding this phenomenon is of great practical importance.

## Mechanisms Contributing to Acute Alterations in Muscle Function after a Voluntary Conditioning Activity: Post-Activation Potentiation Versus Post-Activation Performance Enhancement

When muscles are activated by the nervous system, action potentials are propagated along the motor axon to the nerve terminal where neuromuscular transmission results in the generation and propagation of action potentials on the muscle membrane in a one-to-one ratio. Action potentials on the muscle membrane trigger the release of Ca^2+^ from the sarcoplasmic reticulum *via* ryanodine receptors that open in response to membrane depolarization. Calcium diffuses throughout the myoplasm, interacting with binding proteins and ion pumps. The subsequent binding of Ca^2+^ to troponin C (“Troponin complex” in Figure 3) unblocks binding sites on actin, allowing myosin binding and subsequent contractile force development. Transfer of Ca^2+^ back into the sarcoplasmic reticulum occurs via the sarco/endoplasmic reticulum calcium pump, leading to dissociation of Ca^2+^ from troponin C and subsequent muscle relaxation. Through this process, neural signals are translated into muscular force, although other processes acting at each step in this process can modulate the neural signal-muscle force relationship. It thus follows that the Ca^2+^ concentration-muscle force relationship, i.e., calcium sensitivity, can also be modulated.

### Myosin Light Chain Phosphorylation

The force augmenting effects of “classic” PAP are observed almost immediately as an increase in the peak force and RFD of a twitch contraction (Brown and Von Euler, 1938; Vandenboom et al., 1993; Hamada et al., 2000; Baudry and Duchateau, 2007; Babault et al., 2008). The most commonly cited mechanism underpinning this effect is an increase in calcium sensitivity of the acto-myosin complex caused by phosphorylation of the myosin regulatory light chain (MRLC), a small polypeptide subunit of myosin. This is evidenced by the strong relationship between the magnitude of increase in twitch response and the magnitude of phosphorylation of the MRLC (Manning and Stull, 1979, 1982; Klug et al., 1982; Moore and Stull, 1984; Vandenboom et al., 2013; Vandenboom, 2017). Furthermore, the contractile force of skinned fibers at a given submaximal concentration of calcium (Ca^2+^) is increased, i.e., more force is produced at any submaximal (Ca^2+^) (Persechini et al., 1985; Sweeney and Stull, 1986), when the MRLCs are phosphorylated. The phosphorylation process is catalyzed by myosin light chain kinase (MLCK; see Figure 3), which is activated by Ca^2+^ when the concentration is sufficient. Current findings, such as those of Zhi et al. (2005), who found that tetanic stimulation of mouse extensor digitorum longus muscles increased MRLC phosphorylation 4-fold and twitch force 1.8-fold but was accompanied by only a small force augmentation in MLCK-deficient or knockout mice, are consistent with this. The phosphorylation process has been shown to increase myosin head mobility allowing for the myosin heads to move closer to actin binding sites (Levine et al., 1996, 1998; Alamo et al., 2008, 2015; Brito et al., 2011), increasing the likelihood, and thus rate, of cross-bridge formation (Metzger et al., 1989; Sweeney and Stull, 1990). Evidence that MRLC phosphorylation has no direct effect on myosin motor function when studied in isolation from the thin filament further indicates that this process affects acto-myosin binding kinetics rather than influencing myosin function directly (Greenberg et al., 2009).

It is important to note that peak isometric force cannot be improved if the muscle (or muscle fiber) is fully activated (Persechini et al., 1985; Metzger et al., 1989) because in this case the maximum number of possible cross-bridge attachments already exists, although there is evidence that force generated during maximal eccentric contractions may be enhanced (Brown and Loeb, 1999). It is also relevant that the effect is greater in type II fibers (Moore and Stull, 1984), which have a lower basal Ca^2+^ sensitivity (Gardetto et al., 1989; Metzger and Moss, 1990) and greater MLCK activity than type I fibers (Moore and Stull, 1984). They are therefore particularly susceptible to stimuli that improve calcium sensitivity (Grange et al., 1993). A practical outcome of this is that individuals with a higher proportion of type II muscle fibers (greater type II myosin content) derive a greater PAP benefit, measured as an increase in the peak twitch force (Hamada et al., 2000) [indeed, they have also been shown to exhibit a greater magnitude of voluntary force enhancement, i.e., PAPE (Seitz et al., 2016b)]. In fact, the observation that MRLC phosphorylation has less effect in type I fibers, partly because of their already-higher Ca^2+^ sensitivity (Gardetto et al., 1989; Metzger and Moss, 1990) and low MLCK activity, identifies MRLC phosphorylation in type II fibers as a requirement for optimum contractile function (relative to type I fibers) rather than a unique and beneficial mechanism by which contractile performance can be “enhanced”; thus, the triggering of MRLC phosphorylation may be an essential strategy for optimum muscle function in individuals with higher proportions of type II myosin. Because such effects are easy to evoke through muscle contractions, some researchers have suggested that the potentiated state is the commonest *in vivo* operating state of fast-twitch skeletal muscles (Brown and Loeb, 1998).

Performances in physical activities where type II fibers contribute strongly, such as those requiring high rates of force development or fast muscle shortening speeds, should be improved most when increased MRLC phosphorylation occurs; this includes the twitch contraction used to estimate the magnitude of PAP *in vivo*. However, because phosphorylation is not expected to affect cross-bridge detachment rates, which is the rate-limiting step in cross-bridge cycling, it is also not observed to increase the maximum unloaded shortening speed of muscle fibers (Persechini et al., 1985; Sweeney and Stull, 1990) or whole muscles (Butler et al., 1983; Palmer and Moore, 1989; Decostre et al., 2000; Gittings et al., 2011). Nonetheless, potentiation measured during loaded concentric shortening is often higher than during isometric twitch contractions (Grange et al., 1998; MacIntosh and Bryan, 2002), concentric force potentiation is greater than isometric potentiation for the same level of MRLC phosphorylation (Xeni et al., 2011), and the effect increases at higher speeds of shortening (Caterini et al., 2011). These findings are consistent with the finding that concentric shortening, particularly at faster speeds, is associated with an increased cross-bridge detachment rate and consequent increase in the time in which cross-bridges are in a non-force-generating state (Piazzesi et al., 2007), which would then allow an amplified effect of the increased attachment rate provided through MRLC phosphorylation (Metzger et al., 1989; Vandenboom et al., 2013). Therefore, improvements in force production at high voluntary shortening speeds (i.e., “power” measured at high, but not low, muscle shortening speeds) might be expected to result from post-contraction changes in force production as well as enhancements in maximum, loaded shortening velocity. That is, PAP might consequently enhance high-speed concentric muscle force production and peak muscle shortening speeds.

Despite the large body of evidence linking MRLC phosphorylation to PAP, evidence for this effect in humans is particularly scarce (see Vandenboom, 2017). Also, other mechanisms may play at least some role. Prolonged, high-frequency stimulation of rat soleus muscle, which has a high type I fiber proportion, does not result in meaningful twitch potentiation despite moderate levels of MRLC phosphorylation being observed (Manning and Stull, 1982; Moore and Stull, 1984). Also, increased expression of (skeletal) MLCK in soleus of transgenic mice did not result in PAP even though stimulation-induced phosphorylation of slow and fast MRLCs is induced (Ryder et al., 2007), although these effects might be explicable by factors such as greater muscle fatigue masking potentiation effects. In line with this, a small potentiation response was still observed in MLCK knock-out mice (Gittings et al., 2017). Similar findings have also been observed *in vivo* during voluntary activation, with individuals who display an increased MRLC phosphorylation (~23% increase observed at 7 min post-activity in vastus lateralis) not producing a greater performance in an explosive, dynamic leg extension task than subjects who showed a decrease (~15%) (Smith and Fry, 2007), i.e., the magnitude of PAPE did not appear to be associated with MRLC phosphorylation, although twitch force was not recorded to allow estimation of PAP.

Notably, however, if MRLC phosphorylation is a main mechanism underpinning post-contraction performance improvements in voluntary dynamic activities (i.e., PAPE; as distinct from PAP) such as jumping, running, and cycling, then one would expect the improvements to temporally match the changes in twitch or submaximal tetanic force and RFD. The phosphorylation process occurs rapidly so performance enhancements should be nearly immediate. However, the process is also reversed rapidly by removal of the phosphate group from the MRLC by myosin light chain phosphatase (MLCP; Figure 3A). This results in a quasi-exponential rate of decline in PAP with a rapid decline in the first ~28 s and only a small effect present by ~5 min (Vandervoort et al., 1983; O’Leary et al., 1997; Hamada et al., 2000; MacIntosh and Willis, 2000), as illustrated in Figure 2. Thus, the effect will be relatively short lived. Despite the known time course of PAP, which closely matches the time course of MRLC phosphorylation, the PAPE effect shown in numerous human studies takes at least several minutes to appear (6–10 min) and then may last for >15 min (Figure 2; Bevan et al., 2010; Wilson et al., 2013; Seitz et al., 2014a). This indicates that MRLC phosphorylation is unlikely to be the predominant mechanism driving voluntary performance enhancement after a bout of intense muscular activity (i.e., PAPE). In order to understand the (possibly different) mechanisms influencing PAPE, it is therefore appropriate to consider other acute physiological responses to exercise.

### Increased Muscle Temperature

After single 2- to 20-s tetanic stimulations of intact cat muscles, Brown and Von Euler (1938) observed increases in intramuscular temperature of ~0.3°C that were stated to peak at ~3 min (but appear at ~5 min according to their Figure 3) before slowly returning to baseline. Therefore, changes in intramuscular temperature after tetanic muscle stimulation do not perfectly correspond with the changes in muscle twitch force that are characteristic of PAP (Brown and Von Euler, 1938); changes in muscle temperature probably therefore minimally affect PAP, either *ex vivo* or *in vivo*.

However, changes in voluntary muscular performance (i.e., PAPE) in humans after brief bouts of exercise are observed to be more temporally aligned [e.g., see temporal data in Wilson et al. (2013)], with intense exercise bouts in humans sometimes resulting in peak increases in temperature several minutes after exercise commencement (Saltin et al., 1968; Gonzalez-Alonso et al., 2000). The temperature increase observed by Brown and Von Euler (1938) was partly reduced by blood flow restriction, indicating that at least some of the temperature increase resulted from arterial inflow while the rest is likely to have resulted from increased muscle metabolism; the time required for vascular bed dilation and thus muscle perfusion may explain the post-contraction delay in temperature rise. Nonetheless, greater increases in temperature might be achieved with higher volumes of exercise than those imposed using tetanic stimulation by Brown and von Euler. For example, 3 min of dynamic, voluntary knee extensor activity increased quadriceps muscle temperature by ~0.9°C (Gonzalez-Alonso et al., 2000), which might be considered to represent an approximate maximum that would be achieved with “brief” intense muscle actions such as the conditioning activities commonly used to evoke PAPE. Based on these data, changes in muscle temperature of 0.3–0.9°C might be achievable with brief, intense bouts of muscle work consistent with those used in many studies of PAPE.

Increases in muscle temperature are clearly and equally associated with increases in both rate of force development and shortening velocity in both fast- and slow-twitch muscles/fibers (Ranatunga, 1982; Stein et al., 1982; Elmubarak and Ranatunga, 1984). These data are consistent with cross-bridge cycling rates being strongly influenced by the temperature-sensitive myosin ATPase reaction (Stein et al., 1982; Brenner and Eisenberg, 1986). It is not unexpected therefore that exercise-induced intramuscular temperature increases trigger significant performance enhancements in activities requiring high levels of muscular power output, such as vertical jumping and sprint cycling (~4–6%^o^C^−1^; Bergh and Ekblom, 1979). Also, in handgrip exercise, a 1°C increase in muscle temperature induced by passive warming (water immersion) increased maximal power by 5.1% (Binkhorst et al., 1977), suggesting that a similar increase in performance is observed across tasks. Indeed, the increase in power during voluntary contractions also appears to be velocity-dependent, with increases of ~2%^o^C^−1^ being observed during cycling at 54 revolutions per minute (rpm) but ~10%^o^C^−1^ observed during cycling at 140 rpm (Sargeant, 1987). These data are consistent with the strong effect of temperature on cross-bridge cycling rates. Based on potential temperature changes of 0.3–0.9°C then, one might expect an increase in muscle power of at least 1–5%, but possibly up to 10%, to result from an increase in muscle temperature alone. Given the time course and magnitude of effect, it is therefore possible that increases in muscle temperature contribute to the improvement in voluntary performance after a brief bout of high-intensity exercise (i.e., PAPE). In studies reporting moderate improvements in performance (1–5%; e.g., Yetter and Moir, 2008; Wyland et al., 2015; Karampatsos et al., 2017), changes in muscle temperature could theoretically explain the majority of the performance improvement, and in studies showing improvements of 5–10% (Mina et al., 2014; Dello Iacono et al., 2016; Kummel et al., 2016; Suchomel et al., 2016; Bogdanis et al., 2017), at least some of the change could be associated with temperature changes. Based on this evidence, PAPE experienced after a conditioning activity (that was not preceded by an extensive warm-up period) might be largely, or wholly, explicable by an increase in muscle temperature, particularly when fast rates of force development or muscle shortening are critical to performance of the test contraction. Also, temperature changes might explain velocity-dependent effects, such as the greater increases seen in faster (180°·s^−1^) versus slower (60°·s^−1^) speed knee extensor torque after an isometric conditioning activity (Fukutani et al., 2013). However, the finding that PAPE might be greater in individuals with a higher type II myosin heavy chain percentage (Seitz et al., 2015) cannot be readily explained by a temperature increase since all fiber types tend to show similar temperature dependence.

### Non-Phosphorylation-Dependent Processes Impacting Ca^2+^ Sensitivity

Ca^2+^ sensitivity (defined as an increase in cross-bridge-generated muscle force for a given level of muscle or fiber activation) may also be influenced by processes other than MRLC phosphorylation.

#### Muscle Temperature

Temperature increases have been shown to have variable effects on Ca^2+^ sensitivity, but typically an increase in temperature is associated with a decrease in Ca^2+^ sensitivity (Stephenson and Williams, 1985). This might be related to a reduced organization of the myosin heads relative to the thick filament axis (Malinchik et al., 1997; Xu et al., 2003) such that the heads are aligned closer to the thick filament and thus further from actin. This reduces the likelihood of myosin binding to actin in the presence of Ca^2+^. Nonetheless, the effect of temperature appears to be modest, and is probably not a major factor influencing Ca^2+^ sensitivity when temperature fluctuations are small (e.g., <2°C after brief muscular contractions). Thus, while increases in muscle temperature tend to improve muscle function, such improvements do not result from its influence on Ca^2+^ sensitivity of the acto-myosin complex. Changes in Ca^2+^ sensitivity resulting from temperature increases cannot therefore underpin either PAP or PAPE effects.

#### Muscle pH

Decreases in muscle pH (i.e., accumulation of H^+^ ions), which are likely to occur after a bout of intense muscle contractions, may exert a small negative effect on Ca^2+^ sensitivity (Martyn and Gordon, 1988). This possibly results from an increase in fiber diameter due to alterations in net charge at the surface of the myofilaments (since myofilaments carry a fixed charge, alterations in charge density within the fiber will alter the net charge at the myofilament). This may affect the Ca^2+^ binding constant of troponin C (Stephenson and Wendt, 1984), actin-myosin kinetics (Krasner and Maughan, 1984; Eisenberg and Hill, 1985; Gulati and Babu, 1985), or Ca^2+^ concentration in the space adjacent to the myofilaments (Godt, 1981). Thus, changes in pH will not increase Ca^2+^ sensitivity after conditioning contractions and cannot therefore underpin PAP or PAPE. Nonetheless, it should also be considered that processes (or interventions) that raise pH might allow for PAP or PAPE to be expressed when other processes are triggered that promote them.

#### Muscle Blood Flow and/or Water Content

Increases in blood flow, and subsequently in muscle water, in response to intense exercise may speculatively increase Ca^2+^ sensitivity. This would cause a decrease in ionic strength (i.e., hypotonicity) within the muscle fibers, largely resulting from water movement into the intracellular space during and after intense exercise (Sjogaard et al., 1985). Such changes have been shown to substantially increase muscle fiber force (Edman and Andersson, 1968; Gordon and Godt, 1970; Thames et al., 1974; Mansson, 1989; Sugi et al., 2013) and shortening velocity (Edman and Andersson, 1968; Thames et al., 1974; Edman and Hwang, 1977; Sugi et al., 2015) in both intact and skinned fiber preparations, although the increase in velocity is not always seen in skinned fiber preparations (Thames et al., 1974; Sugi et al., 2013). This effect is believed to result from an increase in the force generated per cross-bridge (Edman and Andersson, 1968; Bressler, 1977; Fink et al., 1986; Sugi et al., 2015; Wang et al., 2015), possibly because the resulting increases in strength of the electrostatic force decrease cross-bridge detachment rates and a subsequently increase working stroke duration (Wang et al., 2015). This would subsequently increase the number of strongly bound, force-producing cross-bridges during dynamic contractions (or force rise leading to stretch of elastic components), assuming that detachment-reattachment rates are unaltered.

The influence of reduced ionic strength is independent of changes in cell volume (i.e., swelling; Gulati and Babu, 1982) and therefore independent of changes in acto-myosin spacing. It is also not likely to be meaningfully influenced by the effect of muscle water on whole muscle passive stiffness, which might otherwise be considered to unload the muscle during shortening and hence contribute to increases in force and shortening velocity (Edman and Hwang, 1977); although this mechanism might provide an additional benefit to muscle force production, as described below (Eng et al., 2018). Of great interest is that this effect has been shown to be greater in mammalian (rat) fast-twitch than slow-twitch fibers (Fink et al., 1986), which is consistent with findings that both PAP and PAPE effects are greater in muscles with greater type II fiber content.

Regardless of the exact mechanism, an increase in force per unit activation (i.e., increase in calcium sensitivity) as a result of reduced intracellular ionic strength (hypotonicity) should enhance muscle function *in vivo*. This effect should, theoretically, have a similar time course to the increases in muscle temperature and blood flow, and thus show a temporal profile sufficient to (at least partly) explain the PAPE effect. Although its effect should emerge too slowly to influence the peak magnitude of PAP *in vivo*, it might theoretically prolong its effects. Given that myofibrillar fluid shifts can increase muscle fiber force, and thus power, in a time course that may be similar to that of PAPE, and that the effect of fluid shifts is fiber-type dependent, this mechanism can plausibly explain PAPE (and general warm-up) effects. The hypothesis that fluid shifts might underpin PAPE should therefore be explicitly tested in future experiments.

### Increased Neural Drive/Muscle Activation

Another possibility is that intense exercise increases the level of voluntary neural drive to the muscle, increasing maximum voluntary RFD and maximal muscle force (Heckman and Enoka, 2012). With respect to voluntary muscle contractions (i.e., PAPE but not PAP), such changes might theoretically result from increases in spinal-level excitability (i.e., increased net motoneuron output), which have been detected after repetitive brief muscle contractions and are associated with increases in muscle activity (measured as electromyography; EMG) (Nuzzo et al., 2016). Nuzzo et al. (2016) found that this increased excitability was detectable immediately after the bout of contractions and slowly decreased to baseline, but not before at least 20 min. Part of this effect may be mediated by changes in arousal level as intense contractions are practiced, since increases in positive arousal are associated with increased muscular force output (Schmidt et al., 2009). It is also of interest that tetanic nerve stimulations in animal models have also resulted in increased excitability of facilitatory (e.g., Ia “stretch”) reflex pathways (Lloyd, 1949; Hagbarth, 1962; Upton et al., 1971), suggesting the possibility that muscle activity may promote acute facilitatory neural adaptations irrespective of aspects of arousal or motivation. *In vivo* in humans, increases in tendon tap and H-reflex amplitudes have also been observed after bouts of intense voluntary muscle contractions (Enoka et al., 1980; Güllich and Schmidtbleicher, 1996; Trimble and Harp, 1998; Folland et al., 2008), although this is not always seen (Wallace et al., 2019). These data indicate that reductions in the inhibitory effects of sensory afferents (through reduced pre-synaptic inhibition) or increases in motoneuron excitability may occur. A brief reduction in the H-reflex response has been observed immediately after muscle contractions (e.g., Trimble and Harp, 1998), although this is expected when H-reflex responses are measured in relaxed (but not active; Xenofondos et al., 2015) muscles due to the effects of post-activation depression and do not indicate a general reduction in excitability of the reflex arc (Hultborn et al., 1996; Tucker et al., 2005). The potentiated H reflex has been observed to gradually return toward baseline, reaching this point at different times across studies (e.g., by 12 min; Folland et al., 2008; although mean H reflex appears elevated), not before 15 min (Güllich and Schmidtbleicher, 1996), or after much longer periods (Trimble and Harp, 1998). Therefore, while such changes might allow for the possibility of an increase in neural drive to the muscle, the different (prolonged) time course compared to the change in voluntary muscle function suggests that other factors must play a more important role. It is also unclear as to how great an influence these changes have on the magnitude of PAPE, especially given that the significant increases in H reflex amplitude documented by Folland et al. (2008) occurred without subsequent increase in voluntary knee extensor function. Finally, it is notable that H reflex assessments have been typically completed in resting muscle rather than during muscular contractions, and such measurements may provide little insight into the effects of intense bouts of exercise on spinal excitability *during* muscle contraction (Tucker et al., 2005).

The use of transcranial (motor-evoked potentials; MEPs) and transmastoid (to evoke cortico-medullary evoked potentials; CMEPs) magnetic stimulations have revealed variable responses depending on many factors, including the intensity and volume of muscular efforts, the time after contraction at which testing was performed, and whether measurements were taken in resting or contracting muscles. A detailed review of these studies is beyond the scope of the current paper, however, by way of example, transient (up to a few seconds) increases in MEP amplitudes have been detected when measured at rest (Nørgaard et al., 2000; Aboodarda et al., 2015; Collins et al., 2017) but not during contraction (Collins et al., 2017) and any increases may be followed by MEP depression (Aboodarda et al., 2015), yet post-activation depression of CMEPs has been reported, even when exercise was sufficiently brief that fatigue should not have been evoked (Aboodarda et al., 2015; Collins et al., 2017). These data, combined with H-reflex data, produce a complex picture of responses of the nervous system to brief muscular efforts.

Nonetheless, based on the findings above, an increase in muscle activation, assessed using EMG and other techniques, might be expected after intense bouts of exercise in at least some studies (i.e., if “neural drive” is enhanced after a conditioning activity). Nonetheless, increases in EMG are usually not observed, even when improvements in functional performance are clearly detected (Hough et al., 2009; Seitz et al., 2015; Mina et al., 2016). In one study, an increase in vastus lateralis EMG was detected at 3 min after performing 2 sets of 5 squats with loads of 25 and 35% or 45 and 65% of maximum load, however a control group (condition) was not included so it cannot be determined whether the effect was a function of the additional warm-up or rather a specific potentiating effect (Sotiropoulos et al., 2010). Regardless, the lack of increase in EMG in most studies might indicate that such increases occur only under specific conditions [e.g., when the movement patterns of the conditioning and test contractions are identical; although this is not the case in Seitz et al. (2015)], or that the EMG technique does not have sufficient resolution to detect small increases in activation of already highly active muscles, especially when processes such as fatigue or potentiation are present (Keenan et al., 2005; Enoka, 2012). However, it must also be considered that these findings indicate that consistent and meaningful improvements in neural drive are not triggered by intense exercise under the conditions adopted in most PAPE experiments. In support of this, in one study the percent voluntary activation (assessed using the interpolated twitch technique) was found to decrease after one to three 10-s maximal isometric knee extensor contractions alongside a significant increase in twitch response (considered to indicate PAP) and without change in voluntary performance (PAPE) (Behm et al., 2004). These data suggest a lack of change in muscle activation after conditioning contractions, and a differential effect on PAP vs. PAPE. Based on current evidence, and despite indications that neural changes may be triggered by voluntary activity, there is a lack of evidence in support of the theory that increases in neural drive to the muscle contribute to increases in complex voluntary muscular performance seen in many studies (i.e., PAPE).

### Increased Muscle-Tendon Stiffness

Both peak twitch and submaximal tetanic forces as well as RFD are strongly influenced by the stiffness of intramuscular series elastic structures (Josephson and Edman, 1998; Edman and Josephson, 2007); thus, outcomes of the currently accepted test of PAP *in vivo* would be affected. This is because the work done by the cross-bridges will be absorbed by elastic tissues as they stretch, storing energy. For example, Edman and Josephson (2007) estimated that 40% of the time to reach 50% of maximum force in single frog muscle fibers (devoid of external tendon) could be attributed to the requirement to stretch series elastic structures – the remaining time was attributable to the excitation-contraction process. In larger and more complex muscles with significant connective tissue mass, the delay in force rise associated with stretching the series elastic component would significantly influence RFD and thus the peak force reached in a brief (e.g., twitch) or submaximal contraction. An increase in stiffness should theoretically promote RFD (in twitch and maximum voluntary contractions) and peak twitch torque, and thus influence the *in vivo* test of PAP.

The tendon and aponeurosis comprise a series elastic tissue continuity that is in series with the muscle itself and should contribute to any series elastic effects, so potential increases in tendon or aponeurosis stiffness might speculatively contribute to force enhancement. However, muscular contractions have been shown, in some cases, to reduce tendon stiffness rather than increase it (Maganaris and Paul, 1999; Kay et al., 2015) and continuing to perform muscular contractions has no additional effect, yet PAP and PAPE have been clearly observed even after numerous maximal pre-conditioning contractions have been performed (Seitz et al., 2014b). Also, a 6-s maximal isometric plantar flexor contraction was observed to evoke a significant increase in twitch force (i.e., PAP) without detectable change in Achilles tendon stiffness in highly trained athletes, even when stiffness was measured at levels of force comparable to the twitch force (Gago et al., 2014). A final piece of relevant evidence is that PAP has been clearly observed in muscles that have very little aponeurotic sheath and from which the tendon has been removed, such as the feline caudofemoralis, an exclusively fast-twitch hip extensor/abductor with a very high fascicle length-to-aponeurosis length (~ 5:1) ratio (Brown et al., 1998; Brown and Loeb, 1999). In such experiments, acute alterations in muscle function must result almost completely from changes within the muscle tissue itself. Thus, based on current evidence, tendon-specific changes are unlikely to underpin either PAP or PAPE effects.

Within the muscle itself, however, changes in parallel or series stiffness could theoretically occur through multiple mechanisms. It is unclear whether intense muscle contractions directly alter the collagen-based connective tissue network within muscles (i.e., the parallel elastic component), although it cannot be discounted that changes such as intramuscular water accumulation might have an impact through hydrogen bonding effects (Zhang et al., 2007). If intramuscular water directly affects collagenous connective tissues, including the aponeuroses, then it should influence (i.e., increase) fiber rotation during contractions. This would have two important effects: (1) to increase muscle shortening velocity for a given fiber shortening velocity (i.e., increase gear ratio) since fiber rotation contributes to muscle shortening, and (2) to increase muscle force by allowing less fiber shortening (and shortening velocity) for a given muscle shortening distance (or velocity). That is, fluid (water)-dependent increases in stiffness may optimize muscle force-velocity and force-length properties during contraction (Eng and Roberts, 2018). However, this hypothesis has yet to be explicitly tested.

A potentially more plausible mechanism is that increases in muscle blood flow and water content trigger the radial bulging of muscles. This has the dual effects of (1) prompting fibers to rotate further during contraction, which would contribute to muscle shortening as described above, and (2) generating an additional, longitudinal passive fiber shortening force as fibers bulge radially while maintaining overall volume. Increases in passive longitudinal forces have been observed, for example, in frog semitendinosus (Edman and Hwang, 1977) and semimembranosus (Sleboda and Roberts, 2017), and have been shown to directly contribute to force and velocity enhancements in active muscle (Edman and Hwang, 1977). A detailed explanation of this phenomenon is outside the scope of the present paper; however, Eng et al. (Eng et al., 2018) have recently provided details as to how increases in muscle fluid pressure might directly influence the force (and velocity)-generating capacity as well as spring-like properties of active muscles. It suffices to conclude, however, that increases in both blood flow and muscle water content subsequent to muscle contraction might meaningfully influence muscle function. Since increases in blood flow occur with increases in temperature, it cannot be discounted that some of the reported temperature-dependent effects on muscular force observed *in vivo* (especially in studies where muscle-based exercise is used to evoke the temperature increase) are mediated through changes in blood flow and, hence, muscle water content. Future research is required to determine the individual versus synergistic effects of these changes.

A final possibility is that Ca^2+^-mediated alterations in titin stiffness might influence force transmission to Z disks, and along fibers. Indirect evidence is provided by the observation of increases in the force generated during rapid muscle stretch after application of a tetanic stimulus (Brown and Loeb, 1999), which are thought to be mediated by titin-dependent mechanisms (Nishikawa et al., 2012; Herzog et al., 2016). Myoplasmic Ca^2+^ transients trigger titin stiffness increases, potentially by a direct increase in the stiffness of the protein itself (although these effects are small) (Labeit et al., 2003; Joumaa et al., 2008b) or through the attachment of titin to actin (Herzog et al., 2016) and the winding effect of actin on titin during sarcomere activation (Nishikawa et al., 2012; Hessel et al., 2017). However, titin stiffness appears to persist for only several seconds after muscles are deactivated (Lee et al., 2007; Joumaa et al., 2008a). Thus, there is no current evidence that the changes can influence the characteristics of muscle contractions performed minutes later.

### Summary

**Figure 4:**
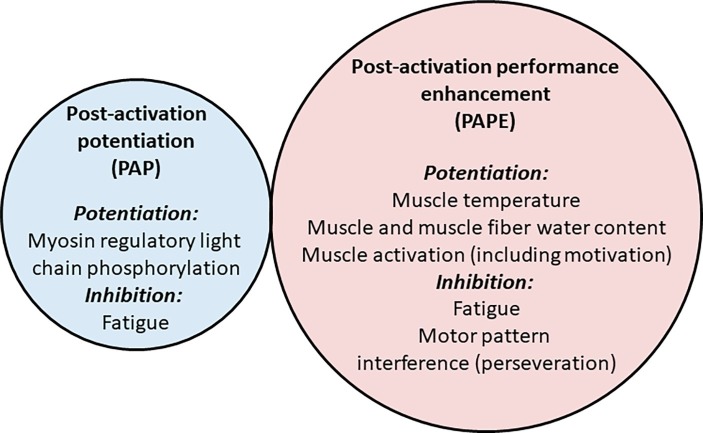
Potential factors influencing post-activation potentiation (PAP) and post-activation performance enhancement (PAPE). The smaller and more transient PAP effect (left circle) is largely explained by myosin regulatory light chain phosphorylation (although other factors may play a small role), and may be briefly inhibited by factors associated with fatigue. The longer-lasting PAPE effect (right circle) may potentially be associated with increases in muscle temperature and muscle water, although enhancements in muscle activation (partly through motivation) may influence performance, while it may be inhibited for several minutes by residual fatigue or motor pattern interference (perseveration) effects. Thus, there may be minimal commonality of mechanism. However, while the mechanisms underpinning PAP are thought to be relatively well defined, there is a lack of evidence regarding the mechanisms that contribute to PAPE.

PAP (defined in the classical sense and tested during a muscle twitch contraction) can be explained largely by myosin light chain phosphorylation, although small contributions from other mechanisms are possible. Nonetheless, PAPE exhibits a different time course, so it is a majoritively different phenomenon. While some residual PAP might influence PAPE in its earliest stages after a conditioning activity, other mechanisms must play a significant role in PAPE (Figure 4). These mechanisms are yet to be defined, although changes in muscle temperature may be important (although they do not seem to explain fiber-type dependence of PAPE), and increases in muscle force and/or shortening velocity triggered by fluid shifts into the working muscles may enhance muscle function in a fiber type-specific manner; these two phenomena might not be mutually exclusive. PAPE appears not to be explicable by an increase in neural drive to the muscle (assessed using EMG and interpolated twitch methods), although some alterations do occur in neural circuitry (e.g., H reflex, MEP and CMEP amplitudes) and changes in motivation/arousal may explain some findings, although the effects of motivation/arousal have not been explicitly tested. Further research is therefore required to completely describe the mechanisms underpinning PAPE, although the majority of the PAP effect has been explained.

## Factors Differentially Influencing Post-Activation Potentiation and Post-Activation Performance Enhancement

The functional role of “classic” PAP has been debated by several authors (Sale, 2002; MacIntosh et al., 2012; Seitz and Haff, 2016; Vandenboom, 2017), with some suggesting positive benefits but others questioning its use in the applied setting. Current evidence shows that MRLC phosphorylation, the most likely phenomenon underpinning PAP, is maximal at low levels of calcium (Sweeney and Stull, 1990). High-intensity muscle contractions such as those used in maximal exercise or heavy lifting (e.g., strength training) require maximal or near-maximal levels of muscle activation, and thus PAP cannot directly influence them. Nonetheless, while effects on maximal force or maximum shortening velocity may be negligible, muscle force output can be impacted through changes in RFD (Sweeney and Stull, 1990; Vandenboom et al., 1993, 1995, 1997) and concentric force production (Grange et al., 1998; MacIntosh and Bryan, 2002). PAP would shift the force-Ca^2+^ relation upward and rightward and therefore force may be augmented at lower levels of activation, such as early in an MVC or in brief contractions where maximal force cannot be reached. This may thus influence performance during intensive, short-duration activities such as jumping or kicking (Sale, 2002). Numerous studies have shown improvements during vertical jumps, for example, after various types of conditioning activities (Duthie et al., 2002; Comyns et al., 2006; McCann and Flanagan, 2010; Crewther et al., 2011; Esformes and Bampouras, 2013; Scott et al., 2017) and this PAPE effect could theoretically be explained, at least in part, as a PAP effect.

However, proof that PAP has underpinned performance enhancement (i.e., PAPE) can only be provided when the outcome variable measured during a voluntary contraction changes with the same time course as the classic PAP response, which is commonly estimated through measurement of the peak muscle twitch force. Unfortunately, few studies have completed both measurements simultaneously, although some authors have at least acknowledged that twitch measurements are needed to verify PAP effects (Hancock et al., 2015). Several researchers have measured changes in twitch force as well as a voluntary force outcome variable in the same or separate sessions in order to examine the magnitudes of change (Behm et al., 2004; Mitchell and Sale, 2011; Kummel et al., 2016; Prieske et al., 2018) or temporal relationships (Gossen and Sale, 2000; Seitz et al., 2014a) between the different outcomes. Findings from such studies do not show that the changes in twitch force are related to the changes in voluntary muscle function, i.e., either PAP was temporally distinct from PAPE (Seitz et al., 2014a; Kummel et al., 2017), PAPE was not observed despite PAP being clearly evoked (Gossen and Sale, 2000; Behm et al., 2004), or few measurements were made and only small increases in twitch force (~10%) were observed at a time point when a (small) performance enhancement was detected (countermovement jump; 2.8% at 4 min) (Mitchell and Sale, 2011). Furthermore, twitch contractile enhancements have been clearly observed without any observable PAPE (e.g., vertical jump; Prieske et al., 2018). Thus, PAP appears to be distinct from PAPE, and the majority of reported incidences of PAPE do not appear to be attributable to PAP. Therefore, it is probably useful to use a different term for each phenomenon in order to clarify which form of potentiation has been evoked. When simultaneous changes in twitch torque and voluntary performance are not shown, perhaps the term PAPE might be used in preference in order to identify the possibility that a separate phenomenon was observed than classical PAP (Cuenca-Fernandez et al., 2017).

One aspect of PAPE that needs further exploration is the lack of effect observed at time points where PAP is strong (e.g., up to 3–4 min post-contraction) since significant PAP should theoretically cause PAPE. The predominant theory for such delay is a coexistence of potentiation and fatigue (Rassier and MacIntosh, 2000; Hamada et al., 2003; Hodgson et al., 2005). A “window of opportunity” has been suggested to define the optimal duration where the potentiation effect remains robust but effects of fatigue have diminished (Docherty and Hodgson, 2007), although others have suggested that it is not possible to generalize recovery durations (Naclerio et al., 2015; Golas et al., 2016). Indeed, recovery duration shows a large inter-individual variability that is associated with numerous factors such as strength level, training experience, and myotypology (Hamada et al., 2003; Naclerio et al., 2015; Golas et al., 2016; Chen et al., 2017).

Nevertheless, while fatigue may influence the force output in the initial stages of PAP (see Rassier and MacIntosh, 2000, for review), evidence of an effect in most studies of PAPE is lacking. Clear increases in twitch force (i.e., PAP) are observed shortly after a voluntary contraction, indicating that fatigue effects are small relative to potentiation effects even in the early stages after contraction (Seitz et al., 2014a). Furthermore, given that only seconds or a few minutes are needed to recover from a short bout of maximal-effort exercise (e.g., less than 1 min for recovery from a maximal squat or bench press lifts or high-intensity cycle ergometer exercise; Hitchcock, 1989; Weir et al., 1994; Matuszak et al., 2003) it is unlikely that a prolonged and substantial fatigue response could act for up to, e.g., 8 min after short-duration conditioning activities, especially given the low volume of activity used in most studies of PAPE. Of course, this does not preclude a role for fatigue when the amount of muscle work is much higher (Vandervoort et al., 1983; Hamada et al., 2003; Xenofondos et al., 2018; Skurvydas et al., 2019). However, it is also likely that other physiological changes augment voluntary muscle function at the time points where PAPE has been observed (i.e., they follow the appropriate time course), such as increases in muscle temperature or coordination (learning or motivational effects), or improvements in muscle function through non-MRLC mechanisms such as intracellular water accumulation.

Another possibility is that the lack of PAPE in the minutes after a conditioning activity, despite PAP being significant, results from a motor pattern interference effect (often called “perseveration” since the motor pattern of one task perseveres while a similar, subsequent task is commenced). This perceived loss of coordination is clearly shown in studies examining the negative influence of sequentially performed tasks (Keele, 1968; Classen et al., 1998; Rubinstein et al., 2001). Broadly, many preceding language, maths, memory, or locomotor tasks can affect the execution speed of a subsequent task, leading to a reduction in performance (Proios and Brugger, 2004; Giannouli, 2013). For motor tasks, Classen and colleagues (Classen et al., 1998) found that the motor cortex may retain specific movement pattern-related aspects of recently practiced movements for many minutes (~20 min in that study). With specific respect to motor tasks, perseveration has typically been observed in simple finger tapping tasks or simulation of lower limb locomotor movement patterns (Keele, 1968; Classen et al., 1998; Gurfinkel et al., 1998), but interference is also clearly demonstrated when force levels produced during a contraction with one mode (e.g., concentric) is performed after several contractions of another mode (e.g., isometric; Kay and Blazevich, 2009) or when one gross motor pattern (e.g., cycling) is performed before another (e.g., running; Gottschall and Palmer, 2002). Nonetheless, perseveration effects have not been explicitly tested in studies of PAPE; so, neither the magnitude of effect nor their temporal profile is known. Given that perseveration effects might better explain the delay in performance enhancement after high-intensity muscular activity than fatigue, and that such effects might be attenuated through use of movement pattern-specific conditioning activities or deliberate task practice subsequent to the conditioning activity, more study is warranted in this area.

## Post-Activation Potentiation and Post-Activation Performance Enhancement Study Design Considerations

If we accept that PAP and PAPE are majorit**i**vely different phenomena and that tests used to describe them are different (i.e., twitch versus voluntary contractions), then it can be concluded that few studies have used the appropriate methodology to reconcile PAP and PAPE. To link PAP and PAPE, important issues related to study design should be considered. As previously discussed, muscle twitch force measurements are considered to reflect the magnitude of PAP, allowing the assessment of the magnitude and time course of PAP in relation to PAPE. Other key points have previously been proposed for the standardization of studies examining either/both PAP/PAPE (MacIntosh et al., 2012), and can be used as a template for a comprehensive list:

1. comparison between at least two conditions; this should include a non-exercising control condition, but should also include an unrelated exercise condition in order to determine whether the conditioning activity was “unique” in its ability to evoke a response, or whether it indicated only an additional “warm-up”;
2. familiarization of the performance task (test) to avoid learning effects;
3. randomization between conditions on separate days;
4. single (researcher) or double (participant + researcher) blinding;
5. control for muscle temperature, time of day, diet and hydration, physical activity performed in the days prior to testing, and potential use of ergogenic aids, should be strongly considered.

A recent study highlighted the impact of some of these points (Cuenca-Fernandez et al., 2017), with the authors observing increases in squat jump height after a non-exercising (i.e., no conditioning activity) control condition, which might indicate a PAP/PAPE effect but could also be attributed to a warm-up (temperature) effect caused by the completion of the baseline tests. In addition, a learning effect inherent to the warm-up may also have also played a key role (Marshall et al., 2019); unfortunately, control conditions (where subjects do not complete the conditioning activities but move immediately to the testing phase) are not always included, so changes in performance cannot always be ascribed to the conditioning activity. Alternatively, torque produced during an MVC might serve as a control condition to exclude some effects of a warm-up, including increases in muscle activation (Nuzzo et al., 2016) or temperature (Bergh and Ekblom, 1979), although increases in peak isometric force might not be particularly temperature sensitive (Rall and Woledge, 1990). Indeed, because PAP mainly influences RFD during a maximal isometric contraction, it is speculatively possible to compare the maximal voluntary force with force produced in very brief, rapid contractions (e.g., <1 s; Duchateau and Baudry, 2014) in cases where twitch force measurements are not possible. However, future research is required to determine whether any improvement in RFD (or muscle power) above maximal voluntary torque is associated with the magnitude of PAP. In addition, it is important that the researchers are blinded to the intervention (conditioning activity; i.e., single blind), and participants should be chosen who are unaware of the possible study outcomes, where possible, so that participant bias is limited (double blinding).

## “Real World” Applicability of Post-Activation Potentiation and Post-Activation Performance Enhancement Research

Based on the outcomes of studies in the area of PAP/PAPE, several points can be raised with regard to real-world validity of experimental designs. One important point is that comprehensive, task-specific warm-up periods appear to have been rarely implemented, which limits the real-world validity of studies in situations where athletes or patients would normally complete an appropriate warm-up routine. In fact, a majority of studies have imposed short-duration, low-intensity warm-up exercises that are insufficient to achieve a complete muscle temperature increase, in particular, or have imposed no warm-up at all (Batista et al., 2007; Berning et al., 2010; Evetovich et al., 2015; Wallace et al., 2019). In studies that imposed a warm-up, routines have typically included light aerobic exercise (generally 5 min of running or cycling at comfortable pace), that could be followed by muscle stretching (several minutes of static or dynamic) and, on some occasions, several repetitions of body-weight resistance exercises or athletic drills (Till and Cooke, 2009; Smith et al., 2014; Wang et al., 2016; Seitz et al., 2016a; Bridgeman et al., 2017; Ulrich and Parstorfer, 2017; Kartages et al., 2019). As one example, increases in sprint running performance were obtained after a conditioning activity consisting of unloaded and loaded sled sprinting (Smith et al., 2014); however, the warm-up consisted of 4 min of stationary cycling. According to best practice, such warm-ups are insufficient to optimize muscle performance, and do not reflect practices of athletes preparing for their activity; although they may be similar to those performed in some clinical environments. It is also possible that long, intense warm-ups might result in critical levels of fatigue, which would reduce the likelihood of further enhancement. Thus, it remains unclear whether the conditioning activities would elicit a meaningful performance enhancement in applied settings where participants would normally complete a comprehensive, task-specific warm-up (pre-exercise preparation) period. This is particularly relevant given that some studies show no PAPE effect in athletes when a test-specific, progressive warm-up is provided (Marshall et al., 2019). In order to assess the potential practical effects of conditioning activities in applied exercise, rehabilitation or sports contexts (i.e., to obtain external validity), therefore, a complete warm-up with task-specific practice should be performed prior to the conditioning activity.

Nonetheless, the current evidence does not discount a PAPE effect even after a full warm-up is completed. In a recent study, countermovement jump height was not found to improve after one set of three back squats with 85% of 1-RM added load when a comprehensive jump-specific warm-up (requiring subjects to be unable to improve jump height over three successive maximal jumps) was performed (Mina et al., 2019). That is, PAPE was not evoked after a comprehensive warm-up even though jump height has been previously shown to be potentiated by prior heavy-squat exercise (see Tillin and Bishop, 2009, for review). Nonetheless, jump height was improved ~6% in another condition when a portion of the 85% 1-RM load was imposed by elastic band resistance in order to alter the magnitude of force applied through the lifting (concentric) phase. This performance enhancement was associated with an increase in force production, knee joint angular velocity, and EMG detected in vastus lateralis in the concentric phase of the jump without change in other kinematic or kinetic variables, suggesting that changes in muscle activation may have underpinned the enhancement. Notably, in this case jump performance did not follow the normal PAPE time course, but instead showed enhancement at all time points measured from 30 s to 12 min. Thus, the possibility exists that performance enhancements can be obtained even after “full” warm-up that includes task-specific practice, although it might show yet a different time course to those shown previously in studies of both PAP and PAPE. Importantly, the inclusion of high-intensity, test-specific muscle contractions in future research studies would allow for determination of whether a chosen conditioning activity might enhance performance more than a “standard,” but comprehensive, warm-up alone; this would be of significant practical use in both athletic and clinical environments.

One issue that will need to be addressed in the future, then, is that of how to judge a “complete” warm-up. For example, several recent studies have reported increases in physical performance in athletes who had previously performed a “sport-specific” warm-up (Feros et al., 2012; Hancock et al., 2015; Munro et al., 2017; Kontou et al., 2018); thus, a functionally relevant PAPE effect was observed. However, in these studies: (1) evidence was not presented to link the improvements specifically to PAP, (2) only a subjective assessment can be made as to whether the warm-ups were “optimum” (appropriate, complete, and with the lowest possible adverse effects, including fatigue), and (3) it was often unclear whether the testing was completed within a reasonable time after warm-up completion (i.e., whether a cool down effect would otherwise have been observed). Importantly, in some of these studies the “standard warm-up” was not described. In other studies, however, various conditioning activities have failed to enhance athlete performance after warm-up (Sarramian et al., 2015; Marshall et al., 2019), and it is not currently clear why results vary between studies. In order to determine whether additional conditioning activities might provide a unique stimulus to the neuromuscular system, i.e., in addition to the continuation or prolongation of the prior (i.e., standard) warm-up, (1) muscle temperature and other physiological measures can be taken, and (2) the “unique” conditioning activity can be compared to one or more other conditioning activities that provide the opportunity for further temperature increase or task-specific practice but are dissimilar to the conditioning activity to be tested (see study design considerations above). Precise details of the warm-up period also need to be included, probably including measurements of exercise intensity and/or effort so that readers can determine for themselves whether the warm-up was appropriate or complete before implementation of the conditioning activity. Regardless, previous findings of performance enhancement in highly trained athletes who are reported to have warmed-up are suggestive that specific conditioning activities might be useful in some contexts. This is an important area of future research.

## Conclusions

The present narrative review examined the time courses of, and mechanisms underpinning, enhancements in muscle twitch properties (demarcating PAP) and voluntary dynamic force production subsequent to a short (acute) bout of high-intensity voluntary exercise (referred to as PAPE). It was confirmed that PAP is a distinct physiological phenomenon with short window of action (seconds to several minutes) that can be largely attributed to myosin light chain phosphorylation within type II fibers and which is observable as an increase in muscle twitch response in skeletal muscle. However, enhancements in voluntary muscular force production, also called PAPE by some authors, was observed as an increase in rate of force development or force during higher speed dynamic contractions, becomes substantive only after several minutes, has a longer window of action than PAP (at least several minutes), and may be largely explained through physiological responses including increases in muscle temperature, intracellular water accumulation, and other mechanisms. PAP and PAPE are often considered to be indistinguishable phenomena, which makes sense from the perspective that many similarities exist, e.g., (1) contractile force is enhanced, (2) some delay exists before potentiation is observed that speculatively results from “fatigue,” and (3) the response is much greater in type II fibers (or muscles with a large fast-twitch fiber proportion). However, other characteristics show them to be largely different phenomena. For example, (1) the time course of their force enhancements differs, with an early effect (within seconds) observed in PAP but delayed effect (after minutes) in PAPE, (2) PAPE but not PAP may be strongly influenced by muscle temperature changes and intramuscular fluid accumulation, and (3) the possibility exists for neural mechanisms to impact PAPE but not PAP. Of note, the current evidence suggests that muscle temperature increases might largely explain PAPE effects under many circumstances; however, it may not explain the greater effect observed in individuals with higher type II fiber content as changes in temperature have similar effects on all mammalian muscle fiber types. Nonetheless, limited evidence *in vivo* in humans but clear evidence *ex vivo* in single fiber studies indicates that increases in intracellular (myocellular) water can enhance muscle force production, and this effect is greater in type II fibers. Further research is therefore warranted in order to examine the relationship between fiber water, fiber type, and muscle function after warm-up exercises (i.e., conditioning activities). Regardless of the exact mechanisms, the current evidence indicates that while PAPE might possibly be affected by PAP in the short time after a conditioning activity, PAPE is rarely observed at time points where PAP is (or is expected to be) maximum, and PAP cannot impact peak muscle force production. The question arises, then, as to whether the triggering of PAP itself has important effects on voluntary muscular force production in most contexts in humans; this question is in need of specific examination. Instead, PAPE might be more practically relevant, and gaining a detailed understanding of the factors impacting PAPE as well as the conditions under which its effects are optimized might be of more practical importance. In this regard, most research reporting PAPE has evoked the response using high-intensity conditioning activities after either no warm-up (physical preparation routine) or a limited/incomplete warm-up, so it is not clear whether it might enhance performance in athlete or clinical populations who perform comprehensive warm-up (or re-warm-up) routines. In cases where a comprehensive warm-up has been reported to occur, variable PAPE responses have been observed and questions remain as to the success of the warm-up itself; specific guidance in relation to experimental design has been provided to determine these outcomes more clearly in future. Despite this, some studies provide encouraging data indicating that PAPE might be evoked by some conditioning activities under some conditions. However, the time course of response does not always match that shown in other PAPE studies, indicating that it might be yet another, partly different, phenomenon or at least that similar physiological changes manifest differently. Further research is required to fully describe both the magnitude of effect and physiological responses underpinning the PAPE phenomenon in order to develop protocols that more reliably evoke the response.

## Author Contributions

AB and NB designed the study, authored the manuscript, and revised and approved the submission.

### Conflict of Interest

The authors declare that the research was conducted in the absence of any commercial or financial relationships that could be construed as a potential conflict of interest.
